# Patterns of species range evolution in Indo-Pacific reef assemblages reveal the Coral Triangle as a net source of transoceanic diversity

**DOI:** 10.1098/rsbl.2016.0090

**Published:** 2016-06

**Authors:** Sean M. Evans, Caroline McKenna, Stephen D. Simpson, Jennifer Tournois, Martin J. Genner

**Affiliations:** 1 School of Biological Sciences, University of Bristol, Bristol BS8 1TQ, UK; 2 Biosciences, University of Exeter, Exeter, EX4 4QD, UK; 3 Centre for Marine Biodiversity, Exploitation and Conservation (MARBEC), Université de Montpellier, CNRS, IRD, Ifremer, Place Eugène Bataillon 34095 Montpellier, France

**Keywords:** biogeography, coral reef, climate change, species distributions, Bayesian skyline plot

## Abstract

The Coral Triangle in the Indo-Pacific is a region renowned for exceptional marine biodiversity. The area could have acted as a ‘centre of origin’ where speciation has been prolific or a ‘centre of survival’ by providing refuge during major environmental shifts such as sea-level changes. The region could also have acted as a ‘centre of accumulation’ for species with origins outside of the Coral Triangle, owing to it being at a central position between the Indian and Pacific oceans. Here, we investigated support for these hypotheses using population-level DNA sequence-based reconstructions of the range evolution of 45 species (314 populations) of Indo-Pacific reef-associated organisms. Our results show that populations undergoing the most ancient establishment were significantly more likely to be closer to the centre of the Coral Triangle than to peripheral locations. The data are consistent with the Coral Triangle being a net source of coral-reef biodiversity for the Indo-Pacific region, suggesting that the region has acted primarily as a centre of survival, a centre of origin or both. These results provide evidence of how a key location can influence the large-scale distributions of biodiversity over evolutionary timescales.

## 1. Introduction

Understanding the causes of spatial distributions of biodiversity is a fundamental goal of contemporary ecology and evolutionary biology. Global-scale analyses of marine diversity show that some locations are unusually rich in species [1] and knowledge of the underlying causes of such patterns can have implications for conservation and sustainable exploitation in a changing world [2]. The Coral Triangle, otherwise known as the Indo-Australian Archipelago, is a region of the Indo-Pacific characterized by coral-rich shelf-seas and a high diversity of reef-associated organisms [3], including over 2000 species of coral-reef-associated fishes [4]. From phylogeographic studies of individual taxa in the region, it is clear that many species show strong spatial genetic structuring, suggesting limited adult dispersal and larval retention [5,6]. Thus, we can largely reject the concept of panmixia within reef-associated species across the region and instead we can consider the relative ages of metapopulations within a species. This provides a useful opportunity to investigate whether specific locations have acted as sources or sinks for populations of individual species.

There is evidence that the Coral Triangle region is a contact zone of taxa for some species that have diversified in allopatry (‘centre of accumulation’, [7]), whereas other studies suggest that it is the source of extant regional diversity, being a ‘centre of origin’ of species [8] and/or a ‘centre of survival’ [9]. In support of the survival hypothesis, there is evidence that the richness of coral-reef fish species, on a global scale, can be best predicted by proximity to reef refugia during the quaternary sea-level changes driven by shifts in global climate [2]. Complementary work using reconstructions of historical biogeography has suggested that the Coral Triangle may have had a changing role through time, where it initially acted as a region of accumulation and survival during the Palaeocene/Eocene, before acting as a centre of origin during the Miocene, and most recently as a centre of survival and export during the Pliocene [10]. Under this model, the region should have acted as a net source of species for the surrounding Indo-Pacific region. Here, we tested if the Coral Triangle has been a net source of extant diversity by reconstructing the historic population sizes of reef-associated species and estimating the relative timing of their population establishments. Specifically, we hypothesized that if the Coral Triangle has acted as a source of extant diversity then populations of individual species in closest proximity to the region should have undergone the most ancient establishments, and there should be an overall reduction in the relative timing of population establishments with increasing distance from the region.

## 2. Material and methods

**Figure 1.**
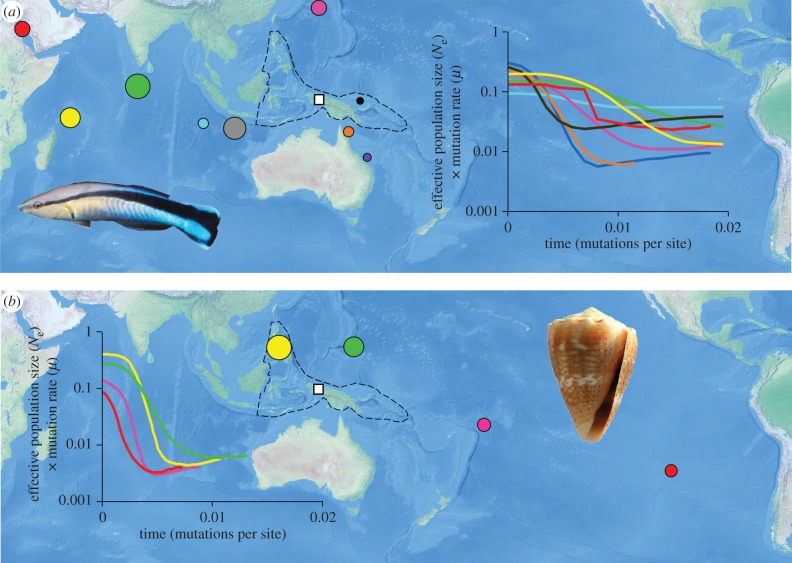
Applications of Bayesian skyline plots to determine relative timing of population establishment: (*a*) bluestreak cleaner wrasse (*Labroides dimidiatus*) and (*b*) thousand-spot cone (*Conus miliaris*). Colours indicate the same populations and circle diameter indicates relative timing of population establishment, also shown on skyline plots. The Coral Triangle ecoregion is highlighted with a dashed line, with the approximate centre indicated with a white square. Indented figures from Brian Gratwicke (https://commons.wikimedia.org/wiki/File:Acanthurus_tennentii_Labroides_dimidiatus.jpg) and Jan Delsing (https://commons.wikimedia.org/wiki/File:Conus_miliaris_001.jpg). (Online version in colour.)

We sourced published population-level DNA sequence data from Genbank (http://www.ncbi.nlm.nih.gov/genbank/) for broadly distributed Indo-Pacific reef-associated fish and invertebrates with a partial distribution in the Coral Triangle region. We used data for species that had sequences from three or more locations, where eight or more individuals had been sampled from each, and where at least one of the locations sampled was within 2500 km of our approximated centre of the Coral Triangle (approximated as 1°35′ S, 135°20′ E, following maps in Green & Mous [3]; figure 1). In total, this yielded data from 45 species, 314 populations in total and an average of 6.97 (range 3 to 23) populations per species (electronic supplementary material, table S1). We aligned data for each species separately using ClustalW in DAMBE v. 5.3 [11], and reconstructed the effective population size through time for each population separately using Bayesian skyline plots in BEAST v. 1.8.2 [12]. Each analysis was run for 10 million steps, using the HKY + *Γ* model. We employed a strict molecular clock and a coalescence Bayesian skyline tree prior with either the default 10 grouped coalescent intervals or instead 4 groups where 10 or fewer individual sequences were available from a location. Operators were set to auto-optimize, and parameters were logged every 1000 iterations. All other search parameters were as default. We aimed to generate only information on the relative timing of effective population size changes, so no temporal calibrations were employed. Bayesian skyline plots were plotted using Tracer v. 1.6 [13]. All populations showed evidence of an expansion towards the present day. Thus, we were able to identify the point in relative time where that constant expansion was initiated [14] and we refer to this as the time of population establishment. Occasionally, populations showed declines in population size after a period of population establishment and growth. Here, we only use the point of the initial population expansion as the time of population establishment.

We estimated the distance of each of the 314 sampled populations to the centre of the Coral Triangle (figure 1). For each species, we fitted a regression line to the relationship between the distance from the centre of the Coral Triangle and the relative time of population establishment. Data for both variables were standardized (mean = 0 and s.d. = 1), ensuring a regression intercept of zero for all species and enabling the calculation of a standardized slope for each species. These slopes were compared to an expected mean slope of zero using a one-sample *t*-test and bias in the direction of slopes was tested using a binomial test. We also tested if populations undergoing the earliest expansions were relatively closer to the centre of the Coral Triangle than those undergoing the latest establishments using a paired *t*-test.

## 3. Results and discussion

**Figure 2.**
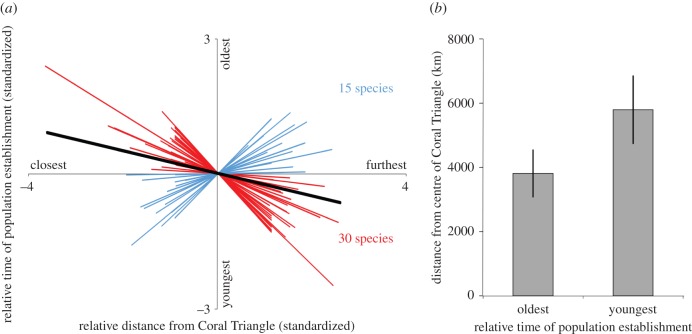
(*a*) Individual species regression lines fitted to relationships between relative distance to centre of Coral Triangle and relative timing of population establishment. Relative times and distances were standardized (mean = 0, s.d. = 1), enabling an average regression slope to be calculated (black line). (*b*) Average geographical distance (±95% CIs) of populations with the earliest and latest population establishment, as estimated from Bayesian skyline plots. (Online version in colour.)

There was a consistent pattern of the populations undergoing earlier establishment (oldest) being closest to the estimated centre of the Coral Triangle (30 of 45 species, binomial test, *p* = 0.010; figure 2*a*, electronic supplementary material, table S2). The slope of the relationship between geographical distance from the centre of the Coral Triangle and the relative time of population establishment was significantly negative (one sample *t*-test, mean slope = −0.215, *n* = 45, *t* = −2.51, *p* = 0.016; figure 2*a*). Populations undergoing the earliest establishments (oldest) were closer to the centre of the Coral Triangle than those undergoing the latest establishments (youngest) (paired *t*-test, *t* = −3.071, *p* = 0.004; figure 2*b*, electronic supplementary material, table S2). Together these results support the hypothesis that the Coral Triangle has acted as a net source of extant Indo-Pacific coral-reef diversity, either by acting as a centre of origin and/or a centre of survival. Notably, time-calibrated phylogenies indicate that Indo-Pacific reef fish species tend to have diverged from their sister taxa on average approximately 3 Ma [15], while intraspecific population divergence and expansion events typically date to within the last million years (e.g. [16]). As such, processes that led to the formation of present day ranges are likely to be decoupled from earlier speciation events, temporally and spatially. Our evidence of the Coral Triangle region acting as a net source of extant species diversity is therefore most parsimoniously interpreted as supportive of the Coral Triangle acting as a centre of survival during Pliocene and Pleistocene environmental changes, including major sea-level fluctuations.

We assumed that populations that have undergone the most ancient establishments are the most likely source of present genetic diversity, whereas those that have undergone more recent establishments are in sink locations. However, we did not explicitly consider genetic interrelationships of populations. Several issues require consideration. First, the extent of genetic connectivity among populations is dependent on life history (duration of larval pelagic dispersal phase) and habitat specialization [17]. Demographic patterns may therefore have become homogenized in some species with gene flow since initial colonisation. Second, phenotypically similar allopatric populations may have persisted in multiple historical refugia, in which case population spread may have been from several geographically segregated sources [18]. Third, studies have demonstrated multiple sympatric clades that may represent cryptic species with overlapping distributions [19], suggesting that finer-scale taxonomic resolution may be required to fully evaluate patterns of population persistence and dispersal. Finally, there is evidence of hybridization among reef-associated species [20], which would make patterns of population establishment difficult to recover. Clearly, therefore, further investigations require accurate dating of the time of population establishments, alongside quantification of the direction and timing of reciprocal gene flow, ideally using information from genome-wide markers [21].

Building on previous studies, our results support the concept that refugia have a pivotal role in the recovery of communities following Pliocene and Pleistocene habitat loss. This emphasizes the importance of refugia for preventing biodiversity loss and has relevance to ongoing threats to shallow water reef communities through habitat destruction, ocean acidification and thermal stress linked to climate change [22]. It has been projected that many species of coral will lose habitat over the next century [23] and the incidence of bleaching will become more frequent [24]. Long-term conservation of tropical reef biotas in a warming world may therefore depend on the identification and preservation of future potential refugia.

## Supplementary Material

Data sources and species-level results summary

## Data accessibility

The supporting datasets are freely available via Genbank (accession numbers in the electronic supplementary material).
