# Supplementary material for: Patterns of species range evolution in Indo-Pacific reef assemblages reveal the Coral Triangle as a net source of transoceanic diversity
Source: Biol Lett. 2016 Jun;12(6):20160090. doi: 10.1098/rsbl.2016.0090 (PMC4938039; doi:10.1098/rsbl.2016.0090)
Supplement: Data sources and species-level results summary [file rsbl20160090supp1.docx]

**Electronic Supplementary Material:** Evans et al. Patterns of species range evolution in Indo-Pacific reef assemblages reveal the Coral Triangle as a net source of transoceanic diversity

**Table S1.** Sources of data used in analyses. Full references are provided below.

| **Class** | **Species** | **Common name** | **Total locations** | **Total sequences** | **Mean sequences per location** | **Data type** | **Reference** | **Genbank Accessions** |
| --- | --- | --- | --- | --- | --- | --- | --- | --- |
| Actinopterygii | *Acanthurus triostegus* | convict tang | 11 | 173 | 15.7 | Control Region | 1 | KJ779749- KJ779871 |
| Actinopterygii | *Amphiprion perideraion* | pink anenomefish | 12 | 182 | 15.2 | Control Region | 2 | JX513647- JX513875; DQ343940 |
| Actinopterygii | *Apogon doederleini* | Doederlein's cardinalfish | 3 | 54 | 18.0 | Control Region | 3 | JF717921- JF717974 |
| Actinopterygii | *Chaetodon auriga* | threadfin butterflyfish | 9 | 239 | 26.6 | Cytochrome b | 4 | KM488667- KM488795 |
| Actinopterygii | *Chaetodon citrinellus* | speckled butterflyfish | 3 | 77 | 25.7 | Control Region | 5 | JX231274- JX231350 |
| Actinopterygii | *Chaetodon lunulatus* | oval butterflyfish | 3 | 79 | 26.3 | Control Region | 5 | JX231351- JX231429 |
| Actinopterygii | *Chaetodon trifascialis* | chevron butterflyfish | 3 | 79 | 26.3 | Control Region | 5 | JX231430- JX231508 |
| Actinopterygii | *Chaetodon vagabundus* | vagabond butterflyfish | 3 | 75 | 25.0 | Control Region | 5 | JX231509- JX231583 |
| Actinopterygii | *Chlorurus sordidus* | daisy parrotfish | 8 | 175 | 21.9 | Control Region | 6 | AY392560- AY392744 |
| Actinopterygii | *Chromis atripectoralis* | black-axil chromis | 3 | 105 | 35.0 | Control Region | 5 | JX231169- JX231273 |
| Actinopterygii | *Chromis viridis* | green chromis | 3 | 90 | 30.0 | Control Region | 5 | JX231584- JX231673 |
| Actinopterygii | *Dascyllus trimaculatus* | threespot dascyllus | 9 | 145 | 16.1 | Control Region | 1 | KJ779398- KJ779534; JF18156- JF18183 |
| Actinopterygii | *Epinephelus fasciatus* | blacktip grouper | 23 | 566 | 24.6 | Cyt b to Control Region | 7 | AB705627- AB706168; AB82990- AB82993 |
| Actinopterygii | *Gobiodon quinquestrigatus* | five-lined coral goby | 3 | 79 | 26.3 | Control Region | 5 | JX231674- JX231752 |
| Actinopterygii | *Halichoeres hortulanus* | checkerboard wrasse | 8 | 137 | 17.1 | Control Region | 1 | KJ779535- KJ779681 |
| Actinopterygii | *Hippocampus barbouri* | Barbour's seahorse | 5 | 96 | 19.2 | Cytochrome b | 8 | AY495716- AY495738 |
| Actinopterygii | *Hippocampus kuda* | common seahorse | 10 | 243 | 24.3 | Cytochrome b | 8 | AY422091- AY422115; AY422126- AY422166 |
| Actinopterygii | *Hippocampus spinosissimus* | hedgehog seahorse | 8 | 170 | 21.3 | Cytochrome b | 8 | AY495739- AY495825 |
| Actinopterygii | *Hippocampus trimaculatus* | flat-faced seahorse | 8 | 83 | 10.4 | Cytochrome b | 8 | AF192699- AF192703; AY322434- AY322476 |
| Actinopterygii | *Labroides dimidiatus* | bluestreak cleaner wrasse | 9 | 116 | 12.9 | Control Region | 9 | KC151127- KC151184; KC151189- KC151207; KC151212- KC151250 |
| Actinopterygii | *Myripristis berndti* | blotcheye soldierfish | 16 | 278 | 17.4 | Cytochrome b | 10 | DQ868393- DQ868429 |
|  |  |  |  |  |  |  |  |  |

**Table S1.** continued

| **Class** | **Species** | **Common name** | **Total locations** | **Total sequences** | **Mean sequences per location** | **Data type** | **Reference** | **Genbank Accessions** |
| --- | --- | --- | --- | --- | --- | --- | --- | --- |
| Actinopterygii | *Naso brevirostris* | spotted unicornfish | 7 | 102 | 14.6 | Control Region | 11 | FJ216727- FJ216828 |
| Actinopterygii | *Naso hexacanthus* | sleek unicornfish | 7 | 86 | 12.3 | Control Region | 12 | KC212823- KC213057 |
| Actinopterygii | *Naso unicornis* | bluespine unicornfish | 5 | 97 | 19.4 | Control Region | 11 | FJ216829- FJ216925 |
| Actinopterygii | *Naso vlamingii* | bignose unicornfish | 8 | 113 | 14.1 | Control Region | 13 | DQ767974- DQ768086 |
| Actinopterygii | *Paracirrhites arcatus* | arc-eye hawkfish | 3 | 77 | 25.7 | Control Region | 5 | JX231753- JX231829 |
| Actinopterygii | *Paracirrhites forsteri* | black-sided hawkfish | 3 | 80 | 26.7 | Control Region | 5 | JX231830- JX231909 |
| Actinopterygii | *Plectropomus leopardus* | leopard coral grouper | 4 | 94 | 23.5 | Control Region | 14 | DQ643415- DQ643465; DQ643476- DQ643519 |
| Actinopterygii | *Plectropomus maculatus* | spotted coral grouper | 7 | 94 | 13.4 | Control Region | 14 | DQ643520- DQ643584; DQ643594- DQ643600; DQ643602- DQ643622 |
| Actinopterygii | *Pseudochromis fuscus* | dusky dottyback | 4 | 93 | 23.3 | Control Region | 15 | AY553417- AY553511 |
| Actinopterygii | *Scarus psittacus* | common parrotfish | 8 | 145 | 18.1 | Control Region | 16. | EU926978- EU927144 |
| Actinopterygii | *Siganus argenteus* | streamlined spinefoot | 3 | 71 | 23.7 | Cytochrome b | 17. | DQ898028- DQ898036 |
| Actinopterygii | *Thalassoma hardwicki* | sixbar wrasse | 8 | 104 | 13.0 | Control Region | 18. | AY185923- AY186029 |
| Actinopterygii | *Zebrasoma scopas* | twotone tang | 3 | 79 | 26.3 | Control Region | 5 | JX231910- JX231988 |
| Actinopterygii | *Zebrasoma veliferum* | sailfin tang | 3 | 73 | 24.3 | Control Region | 5 | JX231989- JX232061 |
| Asteroidea | *Acanthaster planci* | crown-of-thorns starfish | 12 | 90 | 7.5 | COI | 19 | FM174472- FM174513; FM174522- FM174536; FM174561- FM174573; FM202070- FM202075 |
| Bivalvia | *Pinctada maxima* | south sea pearl | 7 | 256 | 36.6 | COI | 20. | JQ990784- JQ990830 |
| Echinoidea | *Tripneustes gratilla* | collector urchin | 7 | 65 | 9.3 | COI | 21 | AY205373- AY205560 |
| Gastropoda | *Conus ebraeus* | black-and-white cone | 6 | 80 | 13.3 | COI | 22 | EF547559- EF547649 |
| Gastropoda | *Conus miliaris* | thousand-spot cone | 4 | 126 | 31.5 | COI | 23 | AY588203; FJ392914- FJ393023; FJ411486- FJ411516 |
| Holothuroidea | *Holothuria whitmaei* | teated sea cucumber | 15 | 352 | 23.5 | COI | 24 | AY176775- AY177134 |
| Malacostraca | *Panulirus homarus* | scalloped spiny lobster | 4 | 202 | 50.5 | D-loop | Unpublished | KM186313- KM186505; KM186511- KM186519 |
| Malacostraca | *Panulirus penicillatus* | red spiny lobster | 9 | 435 | 48.3 | Control Region | 25 | AB689204- AB689672 |
| Malacostraca | *Penaeus monodon* | giant tiger prawn | 9 | 311 | 34.6 | Control Region | 26 | EU426576- EU426831 |
| Reptilia | *Aipysurus laevis* | olive sea snake | 8 | 188 | 23.5 | ND4 | 27 | EF506638- EF506675 |
|  |  |  |  |  |  |  |  |  |

**Table S2.** Relative timing and locations of earliest and latest population establishment within the data for 45 species, and slope of the relationship between standardised time of establishment and standardised distance to centre of the Coral Triangle.

| **Species** | **Location of earliest establishment** | **Time of earliest establishment (scaled by mutation rate)** | **Distance from Coral Triangle centre (km)** | **Location of latest establishment** | **Time of latest establishment (scaled by mutation rate)** | **Distance from Coral Triangle centre (km)** | **Regression slope (relative time of establishment vs relative distance to centre of Coral Triangle)** |
| --- | --- | --- | --- | --- | --- | --- | --- |
| *Acanthurus triostega* | Tuvalu | 0.005 | 4921 | Kavieng | 0.000 | 1720 | 0.318 |
| *Amphiprion peridaraion* | Biak | 0.038 | 94 | Kendari | 0.019 | 1451 | -0.391 |
| *Apogon doederleini* | Lizard Island | 0.016 | 1826 | Ningaloo | 0.009 | 3213 | -0.998 |
| *Chaetodon auriga* | Madagascar | 0.002 | 10117 | French Polynesia | 0.001 | 8352 | 0.015 |
| *Chaetodon citrinellus* | Great Barrier Reef | 0.024 | 1827 | French Polynesia | 0.003 | 8352 | -0.957 |
| *Chaetodon lunulatus* | Great Barrier Reef | 0.005 | 1827 | French Polynesia | 0.002 | 8352 | -0.980 |
| *Chaetodon trifasciatus* | Great Barrier Reef | 0.009 | 1827 | French Polynesia | 0.007 | 8352 | -0.998 |
| *Chaetodon vagabundus* | New Caledonia | 0.007 | 4074 | French Polynesia | 0.005 | 8352 | -0.906 |
| *Chlorurus sordidus* | Lizard Island | 0.024 | 1826 | Seychelles | 0.014 | 8878 | -0.274 |
| *Chromis atripectoralis* | New Caledonia | 0.022 | 4074 | French Polynesia | 0.002 | 8352 | -0.788 |
| *Chromis viridus* | New Caledonia | 0.021 | 4074 | French Polynesia | 0.003 | 8352 | -0.756 |
| *Dasyllus trimaculatus* | Chagos | 0.021 | 7049 | Solomon | 0.007 | 2556 | 0.190 |
| *Epinephelus fasciatus* | Taiwan (south) | 0.003 | 3048 | Kozu | 0.001 | 3981 | -0.562 |
| *Gobidon quinquestrigatus* | Great Barrier Reef | 0.009 | 1827 | New Caledonia | 0.008 | 4074 | 0.177 |
| *Halichoeres hortulanus* | Kavieng | 0.018 | 1720 | Chagos | 0.005 | 7049 | -0.109 |
| *Hippocampus barbouri* | Java | 0.002 | 2998 | Bali | 0.001 | 2380 | 0.759 |
| *Hippocampus kuda* | Bali | 0.003 | 2429 | Thailand | 0.000 | 4120 | -0.263 |
| *Hippocampus spinosissimus* | Cambodia | 0.007 | 3771 | Vietnam | 0.002 | 3269 | -0.216 |
| *Hippocampus trimaculatus* | Cambodia | 0.004 | 3769 | Lombok | 0.002 | 2247 | 0.577 |
| *Labroides dimidiatus* | Maldives | 0.024 | 6889 | PNG | 0.007 | 1480 | 0.526 |
| *Myripristis berndti* | Seychelles | 0.004 | 8878 | Clipperton | 0.000 | 12836 | -0.367 |
| *Naso brevirostris* | Seychelles | 0.063 | 8877 | Cocos | 0.022 | 4407 | -0.167 |
| *Naso hexacanthus* | Seychelles | 0.109 | 8877 | Marquesas | 0.061 | 9471 | -0.415 |
| *Naso unicornis* | Western | 0.069 | 2759 | Seychelles | 0.055 | 8877 | -0.666 |

**Table S2.** continued.

| **Species** | **Location of earliest establishment** | **Time of earliest establishment (scaled by mutation rate)** | **Distance from Coral Triangle centre (km)** | **Location of latest establishment** | **Time of latest establishment (scaled by mutation rate)** | **Distance from Coral Triangle centre (km)** | **Regression slope (relative time of establishment vs relative distance to centre of Coral Triangle)** |
| --- | --- | --- | --- | --- | --- | --- | --- |
| *Naso vlamingii* | Christmas Island | 0.129 | 3422 | French P | 0.074 | 8352 | -0.180 |
| *Paracirrhites arcatus* | French Polynesia | 0.022 | 8352 | Great Barrier Reef | 0.015 | 1827 | 0.933 |
| *Paracirrhites forsteri* | Great Barrier Reef | 0.026 | 1827 | New Caledonia | 0.010 | 4074 | -0.719 |
| *Plectropomus leopardus* | Capricorn | 0.012 | 3018 | Abrolhos | 0.003 | 3759 | -0.104 |
| *Plectropomus maculatus* | Hervey | 0.020 | 3198 | Torres | 0.004 | 1248 | 0.749 |
| *Pseudochromis fuscus* | Kimbe | 0.017 | 1712 | Loloata | 0.004 | 1583 | -0.320 |
| *Scarus psittacus* | Cocos Island | 0.007 | 4408 | Marquesas | 0.003 | 9462 | -0.506 |
| *Siganus argentus* | New Caledonia | 0.003 | 4070 | Truk | 0.001 | 2080 | 0.841 |
| *Thalassoma hardwicki* | Zambales | 0.017 | 1835 | Marou | 0.011 | 2965 | -0.398 |
| *Zebrasoma scopas* | French Polynesia | 0.061 | 8352 | New Caledonia | 0.040 | 4074 | 0.926 |
| *Zebrasoma veliferum* | Great Barrier Reef | 0.038 | 1827 | French Polynesia | 0.027 | 8352 | -0.972 |
| *Acanthaster planci* | Moorea | 0.007 | 8355 | Enderby | 0.001 | 6156 | 0.429 |
| *Pinctada maxima* | Solomon | 0.003 | 2721 | Torres | 0.000 | 1284 | 0.391 |
| *Tripneustes gratilla* | Philippines | 0.004 | 2533 | Easter Island | 0.002 | 12418 | -0.512 |
| *Conus ebraeus* | Philippines | 0.002 | 1724 | Hawaii | 0.001 | 7897 | -0.636 |
| *Conus miliaris* | Philippines | 0.010 | 1724 | Easter Island | 0.005 | 12418 | -0.855 |
| *Holuthuria whitmaei* | Kenn | 0.005 | 3103 | Coral | 0.002 | 3335 | 0.079 |
| *Panulirus homanus* | Vietnam | 0.040 | 3269 | Oman | 0.015 | 8499 | -0.672 |
| *Panulirus penicillatus* | Okinawajima | 0.039 | 3221 | Isabela | 0.025 | 14859 | -0.880 |
| *Penaeus monodon* | North Australia | 0.021 | 1706 | East Thailand | 0.008 | 3979 | 0.020 |
| *Aipysurus laevis* | Hibernia | 0.001 | 1757 | Ashmore | 0.000 | 1794 | -0.056 |
|  |  |  |  |  |  |  |  |

**References in Supporting Information**

1. Liggins L, Treml EA, Possingham HP, Riginos C. 2016. Seascape features, rather than dispersal traits, predict spatial genetic patterns in co-distributed reef fishes. *J. Biogeogr.* **43**, 256-267.

2. Dohna TA, Timm J, Hamid L, Kochzius M. 2015. Limited connectivity and a phylogeographic break characterize populations of the pink anemonefish, *Amphiprion perideraion*, in the Indo-Malay Archipelago: inferences from a mitochondrial and microsatellite loci. *Ecol. Evol.* **5**, 1717-1733.

3. Mirams AGK, Treml EA, Shields JL, Liggins L, Riginos C. 2011. Vicariance and dispersal across an intermittent barrier: population genetic structure of marine animals across the Torres Strait land bridge. *Coral Reefs* **30**, 937-949.

4. DiBattista JD, Waldrop E, Rocha LA, Craig MT, Berumen ML, Bowen BW. 2015. Blinded by the bright: a lack of congruence between colour morphs, phylogeography and taxonomy for a cosmopolitan Indo-Pacific butterflyfish, *Chaetodon auriga*. *J. Biogeogr.* **42**, 1919-1929

5. Messmer V, Jones GP, Munday PL, Planes S. 2012. Concordance between genetic and species diversity in coral reef fishes across the Pacific Ocean biodiversity gradient. *Evolution* **66**, 3902-3917.

6. Bay LK, Choat JH, van Herwerden L, Robertson DR. 2004. High genetic diversities and complex genetic structure in an Indo-Pacific tropical reef fish (*Chlorurus sordidus*): evidence of an unstable evolutionary past? *Mar. Biol*. **144**, 757-767.

7. Kuriiwa K, Chiba SN, Motomura H, Matsuura K. 2014. Phylogeography of Blacktip Grouper, *Epinephelus fasciatus* (Perciformes: Serranidae), and influence of the Kuroshio Current on cryptic lineages and genetic population structure. *Ichthyol. Res.* **61**, 361-374.

8. Lourie SA, Green DM, Vincent ACJ. 2005. Dispersal, habitat differences, and comparative phylogeography of Southeast Asian seahorses (Syngnathidae: *Hippocampus*). *Mol. Ecol.* **14**, 1073-1094.

9. Sims CA, Riginos C, Blomberg SP, Huelsken T, Drew J, Grutter AS. 2014. Cleaning up the biogeography of *Labroides dimidiatus* using phylogenetics and morphometrics. *Coral Reefs* **33**, 223-233.

10. Craig MT, Eble JA, Bowen BW, Robertson DR. 2007. High genetic connectivity across the Indian and Pacific Oceans in the reef fish *Myripristis berndti* (Holocentridae). *Mar. Ecol. Prog. Ser.* **334**, 245-254.

11. Horne JB, van Herwerden L, Choat JH, Robertson DR. 2008. High population connectivity across the Indo-Pacific: congruent lack of phylogeographic structure in three reef fish congeners. *Mol. Phylogenet. Evol.* **49**, 629-638.

12. Horne JB, van Herwerden L. 2013. Long-term panmixia in a cosmopolitan Indo-Pacific coral reef fish and a nebulous genetic boundary with its broadly sympatric sister species. *J. Evol. Biol.* **26**, 783-799.

13. Klanten OS, Choat JH, van Herwerden L. 2007. Extreme genetic diversity and temporal rather than spatial partitioning in a widely distributed coral reef fish. *Mar. Biol.* **150**, 659-670.

14. van Herwerden L, Choat JH, Dudgeon CL, Carlos G, Newman SJ, Frisch A, van Oppen M. 2006. Contrasting patterns of genetic structure in two species of the coral trout *Plectropomus* (Serranidae) from east and west Australia: Introgressive hybridisation or ancestral polymorphisms. *Mol. Phylogenet. Evol.* **41**, 420-435.

15. Messmer V, van Herwerden L, Munday PL, Jones GP. 2005. Phylogeography of colour polymorphism in the coral reef fish *Pseudochromis fuscus*, from Papua New Guinea and the Great Barrier Reef. *Coral Reefs* **24**, 392-402.

16. Winters KL, van Herwerden L, Choat JH, Robertson DR. 2010. Phylogeography of the Indo-Pacific parrotfish *Scarus psittacus*: isolation generates distinctive peripheral populations in two oceans. *Mar. Biol.* **157**, 1679-1691.

17. Lemer S, Aurelle D, Vigliola L, Durand J-D, Borsa P. 2007. Cytochrome b barcoding, molecular systematics and geographic differentiation in rabbitfishes (Siganidae). *C. R. Biol.* **330**, 86-94.

18. Chen CA, Ablan MCA, McManus JW, Bell JD, Tuan VS, Cabanban AS, Shao K-T. 2004. Population structure and genetic variability of six bar wrasse (*Thallasoma hardwicki*) in northern South China Sea revealed by mitochondrial control region sequences. *Mar. Biotechnol.* **6**, 312-326.

19. Vogler C, Benzie J, Lessios H, Barber P, Wörheide G. 2008. A threat to coral reefs multiplied? Four species of crown-of-thorns starfish. *Biol. Lett.* **4**, 696-699.

20. Lind CE, Evans BS, Elphinstone MS, Taylor JJU, Jerry DR. 2012. Phylogeography of a pearl oyster (*Pinctada maxima*) across the Indo-Australian Archipelago: evidence of strong regional structure and population expansions but no phylogenetic breaks. *Biol. J. Linnean Soc.* **107**, 632-646.

21. Lessios HA, Kane J, Robertson DR. 2003. Phylogeography of the pantropical sea urchin *Tripneustes*: contrasting patterns of population structure between oceans. *Evolution* **57**, 2026-2036.

22. Duda TF, Lessios HA. 2009. Connectivity of populations within and between major biogeographic regions of the tropical Pacific in *Conus ebraeus*, a widespread marine gastropod. *Coral Reefs* **28**, 651-659.

23. Duda TF, Lee T. 2009. Isolation and population divergence of a widespread Indo-West Pacific marine gastropod at Easter Island. *Mar. Biol.* **156**, 1193-1202.

24. Uthicke S, Benzie JAH. 2003. Gene flow and population history in high dispersal marine invertebrates: mitochondrial DNA analysis of *Holothuria nobilis* (Echinodermata: Holothuroidea) populations from the Indo-Pacific. *Mol. Ecol.* **12**, 2635-2648.

25. Abdullah MF, Chow S, Sakai M, Cheng J-H, Imai H. 2014. Genetic diversity and population structure of pronghorn spiny lobster *Panulirus penicillatus* in the Pacific region. *Pac. Sci*. **68**, 197-211.

26. You, E-M, Chiu TS, Liu KF, Tassanakajon A, Klinbunga S, Triwitayakorn K, de la Peña LD, Li Y, Yu HT. 2008. Microsatellite and mitochondrial haplotype diversity reveals population differentiation in the tiger shrimp (*Penaeus monodon*) in the Indo- Pacific region. *Anim. Genet.* **39**, 267-277.

27. Lukoschek V, Waycott M, Marsh H. 2007. Phylogeography of the olive sea snake, *Aipysurus laevis* (Hydrophiinae) indicates Pleistocene range expansion around northern Australia but low contemporary gene flow. *Mol. Ecol.* **16**, 3406-3422.
